# American Medical Association

**Published:** 1859-06

**Authors:** 


### EDITORIAL AND MISCELLANEOUS.

### AMERICAN MEDICAL ASSOCIATION.

The American Medical Association met in Louisville, Kentucky, on the 3d of May, but we find the proceedings so utterly devoid of interest, that we think it would be a waste of our space to occupy it by their full publication.—The truth is now palpable—and it is one which we long since announced—that the Association will soon lose all hold upon the interest, respect and confidence, of the mass of the profession, unless the question of medical education is entirely excluded from it. It is a question with which the Association has nothing to do, and over which it cannot possibly exercise any control. Besides, the mass of the profession have really never had anything to do with the *agitation* upon this subject; and so far as the South is concerned as a body, feel no special interest in it. We propose to pass in review at some future period, this whole subject, and present in bold relief, the demagogues and tricksters, whose selfishness and underground intrigues would better become corrupt politicians, than the professed devotees of a noble science, directed to the highest conceivable earthly objects. The wisest proposition, in our judgment, ever submitted to the American Medical Association, and the one fraught with the most good to the permanent prosperity and continued usefulness of that body, was presented at the meeting in Nashville, viz.: To ignore the whole subject of Medical Education, so far at least as any attempt at the exercise of a controlling power, either directly or indirectly, was concerned.

At this same meeting, however, was inaugurated the *present* movement upon this subject; the offspring of envy, jealousy and contemptible personal motives, which is completely absorbing, and displacing what should be the great objects of the Association, the advancement of science and the promotion of harmony in the profession.

We publish below a most sensible and appropriate article from a Louisville paper, in reference to the Association and its objects, past and prospective. The article was evidently written by a medical man, and one who had closely observed the present aspect of medical affairs, as connected with the Association, and its relations to other medical bodies and the profession generally:

NATIONAL MEDICAL ASSOCIATION.—The assembling of delegates from nearly all the organized medical societies and medical colleges in the United States is an occasion of interest not only to physicians but to the public.

Every reflecting mind is naturally led to ask what are the objects of a gathering, involving so much time, trouble, and expense to those directly interested, and every intelligent physician is likely to inquire how far these meetings have been beneficial in their influence upon the profession. The present in the twelfth meeting of this association, and its value as a means of improvement ought therefore at the present time to be well understood·

The objects for which it was organized were the formation of a code of medical ethics, fixing a higher and uniform standard of medical education in the various medical schools, the promotion of medical science, and the interests of the profession in general. In regard to the first of these objecsts, it may be truly said that the action of the association has been eminently successful. The code promulgated cannot fail to be of service. It embodies in concise and simple terms those cardinal rules of conduct which the medical fraternity of every country have uniformly acknowledged as just and wise. Thus far the task was easy.

With reference to the second of these points, the same assertion cannot by any means be maintained. While it is undeniable that the lapse of twelve years has witnessed some improvements in the methods of teaching, it must still be admitted that the requirements for graduation remain essentially the same as they were before. Public opinion may be somewhat more enlightened. Medical schools and medical journals are more numerous, but these changes, whether improvements or not, are hardly traceable to the action of the National Association.

When the cause of failure in a point so important is sought, it may be found in part in the fact that the association is not clothed with any legal power over the subject. Reports, speches, and resolutions without number have been made, but they have not received the force of laws, many of them not even the sanction of public opinion. It may also in part be attributed to the fact that the objects sought were to some extent undesirable or unattainable. Uniformity could scarcely be desired or expected when the circumstances were entirely different.—

One school might have six professors and another eight; one might lecture sixteen weeks, another five months yet both be eminently useful in their respective spheres and each adapted to the means and wants of different classes of students. On this point it may not be deemed amiss to suggest that, since the association is not clothed with any legal authority; since discussion of the subject has awakened jealousies between different schools as well as between the schools as a class and the medical societies; since much time is consumed which is valuable for other purposes; since the colleges are jealous and justly so of their chartered rights, little good can be expected from too urgent demands for action when action is of no avail.

In regard to promotion of medical improvement, a more favorable view of the past and more hopeful view of the future may be indulged. In promoting friendly and scientific intercourse among its members, by encouraging the production of essays by the offer of prizes and otherwise, the Association has done much good, and if it be alleged that much unworthy of its national character has been published under its sanction, that incompetent individuals may sometimes have been clothed with its influence as chairman of its committees, these, it will be admitted, are minor and transient evils, which by no means counterbalance the benefits it has conferred.

But the question may now be fairly put whether the experience of twelve years has not suggested improvements in the method of conducing its business calculated to increase its efficiency and to obviate the evils complained of. It would be strange if it were not so.

A citizen of Louisville who may be led by curiosity to attend its sessions to-day as a spectator will be surprised (unless this should be different from former meetings) to find that these gentlemen brought together from distant States for mutual improvement, scarcely discuss the subjects which relate to the cure of diseases or discourses on science. On the contrary, they sit quietly by while a few of their number debate systems of education as if they were members of a legislative body, propose amendments to the constitution and discuss them with as much gravity as if it were the Constitution of the United States.

We will be still more surprised to learn that scientific reports, prize essays, etc., the contents of which may concern the members personally, are for the most part not read except by title, but are passed to the committee on publication and that the members separate in ignorance to a great degree of what is to be printed with their sanction (in a manner), and in total ignorance of the views of their colleagues on important and undecided points of medical and surgical practice.

The course of this Association is in this respect different from that of almost every similar body. The American Association for the advancement of science is divided into sections to each of which appropriate subjects are assigned. These are discussed, papers read, and reports amended. The advantages of this plan are obvious, it seems indeed so necessary that to propose is to commend it to attention and favor: For the purpose of this Association it is probable that the following arrangement would be as convenient as any. Let it be divided into sections:

1. Anatomy, Surgery, and Physiology.
2. Practice of Medicine and Obstetrics.
3. Chemistry, Botany, and Materia Medica.

Let each meet separately, read, hear, and discuss reports, essays, and papers on the various branches assigned to it. A committee for each section might, after the present meeting, arrange the order of business.

Whether or not these suggestions be carried out, it is greatly to be hoped that a body composed as this is of the great and choice spirits of a learned and humane profession, a body claiming to be national in its character, acting under the eye of an enlightened public, will not forget for a moment the claims of humanity. The science is imperfect and progressive, and it ought to be expected of each member of this great council not only that he will return home better fitted than before to relieve some suffering friend, but also that he should have communicated in exchange some valuable knowledge to his colleagues. If, instead of this, men so eminent are found wasting their time in unimportant discussion, what can be thought but that rivalries, personal objects, and unworthy ambition have usurped the place of humanity and the love of science. To make discoveries, to diffuse knowledge, to apply science to the cure of disease, are truly worthy and dignified objects. If true to those, the American Medical Association is assured of that honor to which it aspires and that sympathy which is a guarantee of success

